# Editorial: The phyllosphere microbiome

**DOI:** 10.3389/fpls.2023.1234843

**Published:** 2023-06-22

**Authors:** Mengcen Wang, Tomislav Cernava

**Affiliations:** ^1^ State Key Laboratory of Rice Biology, and Ministry of Agricultural and Rural Affairs Laboratory of Molecular Biology of Crop Pathogens and Insects, Zhejiang University, Hangzhou, China; ^2^ Key Laboratory of Biology of Crop Pathogens and Insects of Zhejiang Province, Institute of Pesticide and Environmental Toxicology, College of Agriculture and Biotechnology, Zhejiang University, Hangzhou, China; ^3^ School of Biological Sciences, Faculty of Environmental and Life Sciences, University of Southampton, Southampton, United Kingdom; ^4^ Institute of Environmental Biotechnology, Graz University of Technology, Graz, Austria

**Keywords:** phyllosphere microbiome, plant-microbe interactions, sustainable agriculture, microbial ecology, microbiome functioning

The phyllosphere microbiome is composed of microbial communities residing in the above-ground compartments of terrestrial plants, as well as the whole spectrum of biomolecules that they produce (Vorholt, 2012; Berg et al., 2020). Recent technological advances in high-throughput sequencing technologies not only have unveiled the incredible diversity of microorganisms residing on above-ground plant surfaces, including bacteria, fungi, viruses, and archaea (Trivedi et al., 2020), but also have revolutionized our understanding of phyllosphere microbial compositions and dynamics in response to host-related cues, biotic stress, environmental, and Anthropocene-linked factors (Matsumoto et al., 2021; Zhan et al., 2022). Furthermore, various studies that focused on microbial functions have shed light on the roles of phyllosphere microbes in nutrient acquisition, disease suppression, stress tolerance, and plant growth promotion (Fan et al., 2019; Liu et al., 2020; Wang and Cernava, 2020; Xu et al., 2021). By investigating the phyllosphere microbiome, we can gain insights into plant-microbe interactions, ecosystem functioning, and prepare a robust basis for sustainable agricultural practices (Wang and Cernava, 2020; Matsumoto et al., 2022; Liu et al., 2023). For the latter, disease-preventing microbes have recently emerged as a highly promising strategy (Wang and Cernava, 2023). In this Research Topic, current challenges in phyllosphere microbiome research were highlighted, including methodological issues. In addition, functional characterizations of specific microbial taxa were conducted, implications of the environment or Anthropocene on phyllosphere microbial communities were revealed, and their application potential in organic agriculture was emphasized.

Surface sterilization of various plant samples is a commonly adopted processing step for high-throughput sequencing (HTS) approaches as well as for the isolation of endophytic microorganisms. However, the potential impact of different surface sterilization techniques on the composition and diversity of endophytes has remained largely overlooked. Yu et al. investigated the influence of sodium hypochlorite (NaClO), a commonly applied disinfectant for sample pre-treatment, on the diversity of endophytic bacteria and fungi in leaves and stems of tea plants. They found that diversity assessments of bacterial endophytes were significantly affected by certain NaClO concentrations as well as the treatment time. In contrast, the diversity of fungal endophytes was not significantly impacted according to the results obtained with HTS. Their results demonstrated that pre-treatments with NaClO should be carefully implemented in phyllosphere microbiome studies, especially in the case of precise assessments of bacterial diversity in different above-ground tissues.

The phyllosphere microbiome contributes to essential functions of host plants, such as defense against various phytopathogens. In one of the articles, the defensive function of an indigenous phyllosphere bacterium from healthy citrus plants against Huanglongbing (HLB), a devastating disease of citrus crops caused by *Candidatus* Liberibacter asiaticus (CLas), was studied by Munir et al. They found that CLas density in citrus trees respond inversely proportional to the density of the beneficial phyllosphere bacterium. Its application on diseased citrus leaves reduced pathogen density in leaf midribs by 1,000-fold *in vitro*. This disease-suppressive phyllosphere bacterium can efficiently colonize the phloem of citrus leaves, which may offer more opportunities for future management of vascular pathogens. Further explorations will be required to decipher disease-suppressive mechanisms of highly active phyllosphere bacteria.

Permafrost mounds in subarctic palsa mires are thawing due to climate change and becoming a substantial source of N_2_O emissions. However, phyllosphere microbiome-related implications were not investigated so far. Nie et al. found that the N_2_O emission potential in palsa bogs is increase by certain phyllosphere bacteria of two dominant *Sphagnum* mosses; especially by such that are found on *Sphagnum capillifolium*. *Pseudomonas* and members of the bacterial family Enterobacteriaceae were identified as pH-responsive hyperactive N_2_O emitters. Their study revealed previously unknown hyperactive N_2_O emitters associated with *Sphagnum capillifolium* that naturally occurs in melting palsa mounds, and provided novel insights into the importance of the phyllosphere microbiome in the Anthropocene.

Organic matter inputs are used as a common agricultural practice to improve soil fertility and quality, but it is sparsely understood how this practice affects the plant microbiome. Köberl et al. investigated microbial colonization of the East African highland banana cultivar “Mpologoma” under different mulch and manure treatments on three representative smallholder farms in Uganda. They found that the gammaproteobacterial community appeared stable with no significant response to organic matter inputs after 24 months of treatment. However, significant differences in the plant’s carpo-, phyllo-, and rhizosphere microbial community composition and diversity were found among individual farms, independent of the added soil inputs. Their results highlight short-term microbial stability in banana cropping systems in terms of a high resilience of gammaproteobacterial communities. However, future investigations that include whole bacterial as well as multi-kingdom communities are required to provide detailed insights into potential impacts of soil amendments on the banana microbiome.

Application of conventional chemical fungicides is a common measure to counteract phytopathogens in agricultural production systems, but can also adversely affect food safety and cause environmental pollution. In the study by Sun et al., the effects of ozonated water, mancozeb, and thiophanate-methyl on the structure, composition, and diversity of strawberry phyllosphere microbial communities were investigated by high-throughput sequencing. Ozonated water was found to efficiently reduce the relative abundance of potentially pathogenic fungi and is therefore a potential biocide that could reduce environmental pollution in the future in contrast to conventional chemical agents. They also found that microbial communities in the strawberry phyllosphere showed a specific response to the employed fungicides. The observed, chemical-specific response may serve as a basis for the implementation of the phyllosphere microbiome as a highly-sensitive indicator for the use of agrochemicals and thus provide an alternative approach to available analytical approaches for residue detection.

The other six studies published in this Research Topic have provided new insights into mechanisms of phyllosphere microbiome assembly and responses in different host plants, including barley, citrus, halophytes, rice, and wheat. They not only increased our understanding of phyllosphere microbiome functions, but also provided new clues for the development of phyllosphere microbiome-based applications that could increase sustainability in agriculture. It can be expected that the required knowledge base that is needed to develop such applications will substantially grow during the next years. Continuous improvements in sequencing technologies as well as data processing will enable a more comprehensive characterization of the phyllosphere microbiome at a finer taxonomic and functional resolution. Integration of multi-omics approaches will provide a deeper understanding of microbial interactions and metabolic networks within the phyllosphere. These advancements will allow us to uncover the hidden diversity and functional potential of the phyllosphere microbiome. They will also enhance our understanding of the intricate interplay between the phyllosphere microbiota and host plants; this also includes the apparent crosstalk with the rhizosphere microbiome. Collectively, it will be important to decipher functional complementarity and synergistic effects between plants and microbes that contribute to plant health and growth, enabling the development of targeted strategies to secure global food production.

## Author contributions

MW prepared the first draft of this editorial and TC conducted a critical revision. All authors contributed to the article and approved the submitted version.
